# A soil database from Queretaro, Mexico for assessment of crop and irrigation water requirements

**DOI:** 10.1038/s41597-023-02332-7

**Published:** 2023-07-04

**Authors:** Nami Morales-Durán, Sebastián Fuentes, Carlos Chávez

**Affiliations:** 1 Chemical Sciencies Faculty, Autonomous University of Nuevo Leon, San Nicolas de los Garza, 66451 Nuevo Leon Mexico; 2 Research Center for Biotechnology and Nanotechnology, Chemical Sciences Faculty, Research and Technological Innovation Park, Autonomous University of Nuevo Leon, Apodaca, 66629 Nuevo Leon Mexico; 3 Water Research Center, Department of Irrigation and Drainage Engineering, Autonomous University of Queretaro. Cerro de las Campanas SN, Col. Las Campanas, 76010 Queretaro, Mexico

**Keywords:** Hydrology, Agriculture, Water resources

## Abstract

Several studies have assessed crop water requirements based on soil properties, but these have been on a small scale or on soils with similar textures. Here, a data base of soil measurements in the field and laboratory from sites across Irrigation District 023, San Juan del Rio, Queretaro, Mexico was sampled, collected, analyzed, and integrated. The data base, named, *NaneSoil*, contains information on 900 samples obtained from irrigated plots. *NaneSoil* cover 10 of the 12 textural classes with the following information: sand, silt, clay contents, bulk density, saturated volumetric water content, field capacity, permanent wilting point and saturated hydraulic conductivity. The aim of this work is to provide the scientific community with sufficient information to perform a large number of analyses, for example, development of pedotransfer functions, calculation of water requirements of plants in soils with similar characteristics, modeling of infiltration, optimal irrigation discharge calculation, among others. The dataset also promotes the scientific community to contribute their own measurements to further strengthen the knowledge of flow in the porous medium.

## Background & Summary

The crop water requirement calculation is of critical importance to meet the demands at different stages, and is sometimes performed using parameters and constants that are obtained from the literature due to lack of on-site measurements^1–3^. According to the U.S. Department of Agriculture (USDA) textural triangle the soil can be classified into 12 different textures according to the percentages of sand (Sa), silt (Si) and clay (Cl) in each soil sample^4^. The water infiltration rate in each type of soil is completely different and knowing its value is of fundamental importance for the correct design of pressurized irrigation systems^5–8^ or the optimal irrigation discharge calculation to be supplied in each border or furrow^9–14^.

For the knowledge of some soil parameters such as saturated moisture content (θ_s_) and saturated hydraulic conductivity (K_s_) can be obtained by field and laboratory tests, but they are costly and time consuming, also, they can be estimated by pedotransfer functions^15,16^ or neural networks^17–19^ that relate more properties of the soil being analyzed. However, to achieve the above, sufficient data is required to have a representative equation of the soil being analyzed. In addition, studies differ in their results, as an example: they make use of parameters that are not normally easy to obtain: pH, cation exchange capacity (CEC), organic matter content (OM), among others, and the results obtained are different with each of the equations being analyzed^20–22^.

Soil samples were collected from plots adequately prepared prior to seeding, located in the Irrigation District 023, San Juan del Rio, Queretaro during the period 2016–2018, where 20% maize (*Zea mays* L.), 15% sorghum (*Sorghum vulgare* Pers.), 10% wheat (*Triticum aestivum* L.), 15% oats (*Avena sativa* L.), 5% beans (*Phaseolus vulgaris* L.), 15% barley (*Hordeum vulgare* L.), 5% carrots (*Daucus carota* L.) and 15% alfalfa (*Medicago sativa* L.) have been traditionally sown. The integration of all soil information was performed in a dataset named *NaneSoil*^23^. The information contained in the dataset can be used to perform statistical analysis, to develop pedotransfer functions^19,24^, use of neural networks^19,25^, calculation of crop water requirement as a function of soil texture^26–28^, surface irrigation system design^29^ and pressurized^30^, or any other agronomic parameter of interest. *NaneSoil* contains most of the agronomic parameters necessary for the study of crop water requirements in different soils, and therefore, it is an excellent tool that can be used by farmers, academics, students, and people involved in crop production and research.

In addition, there are different databases worldwide such as Harmonized World Soil Database (HWSD)^31^ or SoilGrids^32^. However, they have a spatial variability ranging from 250 m to 1000 m per pixel side, which means that even plots of different textures are taken as one. Whereas this work has a resolution of 100 m per pixel side. In addition, they also do not work with field capacity moisture, permanent wilting point or K_s_, which is relevant for the calculation of crop requirements.

## Methods

### Data collection

**Fig. 1:**
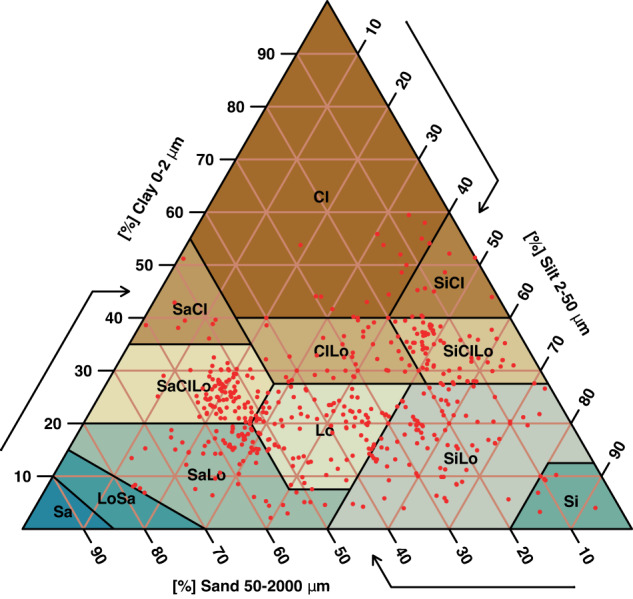
Spatial distribution of the textures contained in the dataset.

*NaneSoil* contains information from 900 soil samples (Fig. 1) that have been classified into 10 of the 12 existing soil textural classes (Table 1). The parameters measured were K_s_, θ_s_, field capacity (θ_FC_), permanent wilting point (θ_PWP_), bulk density (BD), Sa, Si, and Cl.

**Table 1:** Soil textures included in *NaneSoil*.

TEXTURE	SAMPLES	AREA (ha)
CLAY (Cl)	30	82.86
CLAY LOAM (ClLo)	64	199.45
LOAM (Lo)	106	364.11
SANDY CLAY (SaCl)	10	35.22
SANDY CLAY LOAM (SaClLo)	140	529.29
SANDY LOAM (SaLo)	108	359.69
SILT (Si)	136	268.06
SILT LOAM (SiLo)	150	399.16
SILTY CLAY (SiCl)	23	65.96
SILTY CLAY LOAM (SiClLo)	133	400.70

Soil samples were collected over a 3-year period and analyzed in the laboratory following conventional methodologies. K_s_ was obtained in the laboratory using the variable head permeability, BD using the cylinder of known volume or core method^33,34^, θ_s_, θ_FC_, and θ_PWP_ by the pressure membrane pot^35^, Sa, Si, Cl were obtained by the granulometric curve using mesh analysis and the Bouyoucos hydrometer^36^ and were classified according to the United States Department of Agriculture (USDA) system. Sa has a particle size ranging from 0.050 to 2.000 mm, Si from 0.002 to 0.050 mm, and Cl less than 0.002 mm. For the laboratory measurements, six subsamples were randomly collected from each plot at a depth of 0–30 cm. The depth is determined by crop type (root development) to manage fertilization, based on soil nutritional supply, crop water demand and that most if the root mass is located in the first 30 cm for the main crops present in the Irrigation District 023^37^. Also, it should be noted that the specified depths are calculated after removal of undecomposed organic residues. There is no standard number of subsamples to be taken in each sampling unit, however, in this dataset the descriptive statistics of the areas of the plots analyzed were considered, which are mean = 2.65 ha, standard deviation (SD) = 1.6099 ha and coefficient of variation (CV) = 0.5357 with which it was decided to set at six subsamples per plot. With this method each sample or soil property has the same probability of being collected and included. In addition, the six subsamples were grouped until a seventh homogeneous sample was obtained from which 1 kg was taken according to the Mexican Official Standard-021-SEMARNAT-2000^35^.

### Data processing

**Fig. 2:**
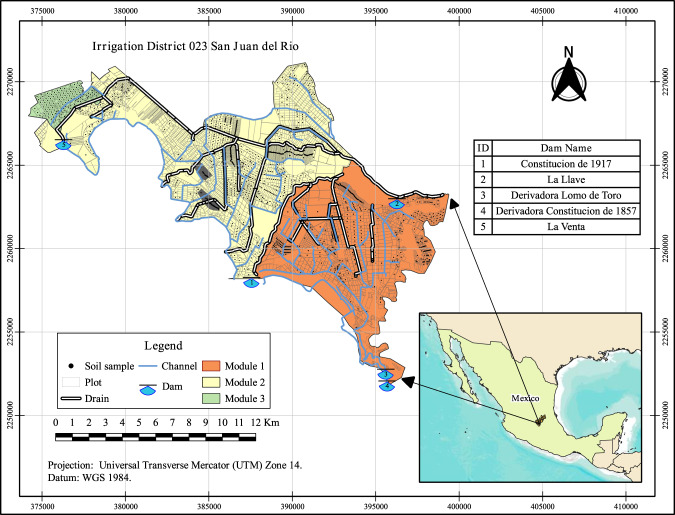
Spatial distribution of soil sample collection sites.

**Fig. 3:**
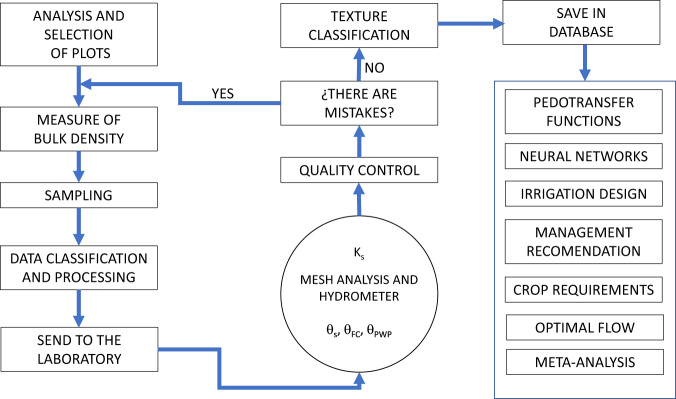
Detailed diagram of the collection procedure, data integration and potential uses of the dataset.

The data processed in each of the laboratory tests were classified by textural class using a worksheet. The total samples were randomly collected from an area of 5,000 ha (Fig. 2). A total of 2,704.5 ha were sampled, and the process followed to obtain the information is shown in general terms in Fig. 3. Sampling and handling in the laboratory were independent.

## Data Records

The dataset^23^ can be downloaded from the Figshare Platform (10.6084/m9.figshare.22190185.v2). The downloaded file *NaneSoil.xlsx* includes 901 rows and 13 columns. The first row corresponds to the titles or attributes contained in the columns, as well as the units in which they are displayed. The thirteen columns are ordered as follows: sample identifier (A), UTM coordinates corresponding to zone 14 North (B and C), representative sample area (D), percentages of sand (E), silt (F) and clay (G), textural classification (H), bulk density (I), saturated moisture content (J), field capacity (K), permanent wilting point (L) and the saturated hydraulic conductivity (M). The USDA texture triangle was used for texture classification.

## Technical Validation

Quality controls were performed on each of the experiments carried out in the laboratory, as well as on the samples taken in the field to verify the fidelity of the data obtained. Each of the samples taken in the field was labeled and carefully transferred to the laboratory for analysis. The label on each sample provided the UTM coordinates (Zone 14 North) from which the samples were collected. Special care was taken to place the samples in the corresponding containers before starting the measurements with the equipment to be used. Before starting the laboratory analysis, the samples were checked to ensure that they had not been previously processed by means of their ID to avoid duplication. At the end of the tests, the results were carefully analyzed and if there were any uncertainties about them, such as BD > 2.65 g/cm^3^ or the sum of the proportion of Sa, Si, Cl > 100%, the results were discarded and the collection of the sample from the field was started again, assigning it another ID for reprocessing (see Fig. 3).

All laboratory equipment used was previously calibrated according to the standards before starting the measurements, and it was checked that it was working in optimal conditions before taking the measurements of the next sample. After entering the data into the dataset, another person checked the records once again to make sure that the data were written correctly. After recording, the format of each column (numerical or string) was checked to correct any typing errors in the columns (e.g., soil texture and UTM coordinates where the samples were collected).

The textural classification was inspected in the texture triangle to ensure that its classification corresponded to the percentages of sand, silt and clay reported. In addition, a visual inspection of the distribution of the data in the columns containing numerical values (e.g., saturated moisture content, permanent wilting point, field capacity, saturated hydraulic conductivity) was performed, and outliers were manually verified by validating them with the original values written in the data logs during the development of the experiments.

## Usage Notes

The soil sample analyses reported in the *NaneSoil* dataset were extracted from agricultural plots that have 2 crop cycles per year: spring-summer and autumn-winter, in which conventional preparations are made before, during and after each harvest. Saturated moisture contents, field capacity and permanent wilting point are reported as volumetric moisture contents. The user may contact the corresponding author for more information about the dataset and its use.

## Data Availability

All the data processing and data visualization were conducted using R (version 4.2.2)^38^. The source code is available on GitHub (https://github.com/Chagcarlos/NaneSoil_Figs). The Code contains detailed comments for the development of Fig. 1 related to the texture triangle. The map presented in Fig. 2 contains the soil sampling locations and was made using QGIS software version 3.16 (https://qgis.org/en/site/forusers/download.html) under GNU General Public License (CC BY-SA 3.0).
